# The Money-Value Again

**Published:** 1892-09

**Authors:** 


### THE MONEY-VALUE AGAIN.

In our July issue we had something to say about the "Money-value of Medical Attendance," and gave the history of an illustrative case which occurred in this city and attracted a good deal of attention and comment outside of it.

Now, there comes to us from the Windy City a story of a similar case. Dr. Waxham, of Chicago, whose name will be familiar to many of our readers from his contributions to the treatment of laryngeal stenosis by intubation, performed this operation on a child suffering with diphtheria, the son of a gentleman many times a millionaire. It was believed that the intubation saved the child's life. The parents were very grateful, and in the exuberance of their gratitude they invited Dr. Waxham to send in his bill. This he promptly did to the extent of $2,000. To accompany it he also sent an explanatory and apologetic note, closing by saying that if the bill seemed a little too large, he would gladly reduce it; that, on the other hand, if the happy father felt like paying more it would be accepted, with the consciousness that the service was worth it. The grateful father was horrified at the size of the bill and vigorously objected. He estimated the services rendered to be worth about $400 and sent his check for that amount. This Dr. Waxham returned with thanks and the suggestion that he either pay the full amount at once, or arbitrate the matter, or have it decided in the courts. An arbitration committee decided that the "sum of $2,000 was a reasonable and proper and just compensation in money for services rendered," and the matter ended with the payment of the bill.

Without passing judgment on the reasonableness or unreasonableness of this fee, we can certainly say that Dr. Waxham made a mistake in sending any note to accompany the bill when first presented. This weakened his position very much. The statement of the account should have been presented without comment, just as if this were the usual fee for same services rendered. Then, if objections were raised, would have been the time for discussions.

